# Why intergroup variation matters for understanding behaviour

**DOI:** 10.1098/rsbl.2019.0695

**Published:** 2019-11-13

**Authors:** Stephan P. Kaufhold, Edwin J. C. van Leeuwen

**Affiliations:** 1 Department of Cognitive Science, University of California San Diego, 9500 Gilman Drive, La Jolla, San Diego, CA 92093, USA; 2 Behavioral Ecology and Ecophysiology Group, Department of Biology, University of Antwerp, Universiteitsplein 1, 2610, Wilrijk, Antwerp, Belgium; 3 Centre for Research and Conservation, Royal Zoological Society of Antwerp, K. Astridplein 26, 2018 Antwerp, Belgium; 4 Max Planck Institute for Psycholinguistics, Wundtlaan 1, 6525 XD Nijmegen, The Netherlands

**Keywords:** intergroup variation, chimpanzees, species-typical behaviour, group differences, culture

## Abstract

Intergroup variation (IGV) refers to variation between different groups of the same species. While its existence in the behavioural realm has been expected and evidenced, the potential effects of IGV are rarely considered in studies that aim to shed light on the evolutionary origins of human socio-cognition, especially in our closest living relatives—the great apes. Here, by taking chimpanzees as a point of reference, we argue that (i) IGV could plausibly explain inconsistent research findings across numerous topics of inquiry (experimental/behavioural studies on chimpanzees), (ii) understanding the evolutionary origins of behaviour requires an accurate assessment of species' modes of behaving across different socio-ecological contexts, which necessitates a reliable estimation of variation across intraspecific groups, and (iii) IGV in the behavioural realm is increasingly likely to be expected owing to the progressive identification of non-human animal cultures. With these points, and by extrapolating from chimpanzees to generic guidelines, we aim to encourage researchers to explicitly consider IGV as an explanatory variable in future studies attempting to understand the socio-cognitive and evolutionary determinants of behaviour in group-living animals.

## 1. Introduction

Within the order of primates, humans are the species occupying the widest range of habitats, spanning from small-scale societies in subarctic climates to cities with millions of inhabitants in desert environments. Correspondingly, humans show a wide range of behavioural proclivities—stemming from the peculiarities of both their physical and social environments—which deems ‘flexibility’ a core characteristic of the human species [1]. In other words, disregarding the existence of behavioural variation on the individual and group levels would inevitably lead to an impoverished view of human nature.

The importance of considering cross-cultural variation for understanding universals and diversity in human cognition and behaviour has gained increasing traction over the past years (e.g. [2–4]). For instance, prosocial [5] and conformist [6] tendencies, as well as norms regarding what constitutes a ‘fair’ division of resources [7], differ markedly across societies. Several mechanisms have been identified underlying the cross-societal diversification of behavioural tendencies, e.g. genetics, environmental affordances, culture [1,8,9] and even gene–culture coevolution [10]. Intergroup variation (henceforth: ‘IGV’) is becoming an integrated level of explanation of behavioural diversity in the human species, especially with regard to social behaviour. The *Proceedings of the National Academy of Sciences* (*PNAS*) recently even published a special issue on the ‘pressing questions in the study of psychological and behavioral diversity’, emphasizing in their introductory article that: ‘A researcher who relies on just one of these [intraspecific] groups to develop and vet a theory of human psychology would have a challenge determining what is basic, fundamental, or universal and what is rather particular to the cultural and social context in which it is being studied’ [11, p. 11367].

Here, we wish to argue that it is reasonable to extend this position to non-human animals (henceforth: ‘animals’). Already in the same *PNAS* issue, there is one study highlighting that groups of chimpanzees differ from one another in their social dynamics, despite experiencing similar socio-ecological conditions [12]. More generally, in the light of the increasing evidence suggestive of the presence of group differences in the social behaviour of animals (e.g. [13–16]), we believe a similar cautioning—i.e. against the implicit assumption that individuals of the same species share a uniform psychology—is justified for the study of animal behaviour. By taking chimpanzees (*Pan troglodytes*) as an illustrative case, we argue that (i) IGV in animals is sufficiently documented to be taken seriously, (ii) its presence could plausibly resolve several scientific controversies, and (iii) without estimating the magnitude of IGV, we are premature in drawing species-typical conclusions. Lastly, we outline a pragmatic protocol for constructively incorporating the effects of IGV in animal studies.

## 2. What is intergroup variation and how does it emerge?

While animals of the same species have many traits in common, every single individual is also marked by distinct features with regard to both its genotype (with the exception of clones) and phenotype. This variation between individuals within the same species is referred to as intraspecific variation [17] and can be the result of both ultimate (genetic variation, developmental plasticity [8]) and proximate (ecology, learning) processes (e.g. [18,19]). However, variation within a species does not only occur on the level of individuals but is also possible on the level of populations and groups. IGV refers to variation between different communities of the same species (e.g. [14,20,21]. Thus, IGV is not observable in an isolated individual, but is a group-level phenomenon that comprises traits that show some stability within one group but can vary across other groups. Given its group-level nature, typically, IGV is spurred by a homogenizing force, for instance, a differential set of ecological affordances (e.g. availability/accessibility of food resources) and/or social determinants like group size and learning biases, most prominently within-group conformity [22–24]. Thus, variation between animal groups of the same species can arise through differences in ecological and/or demographic conditions [25], but also through socio-cognitive mechanisms [12,24].

Here, we will primarily focus on group-level variation in social behaviour and elucidate how its existence could possibly account for controversies across experimental studies on great ape behaviour and cognition (e.g. cooperation [26–29], prosociality [30–36] (van Leeuwen EJC, DeTroy SE, Kaufhold SP, Dubois C, Schütte S, Call J, Haun DBM 2016, unpublished manuscript) and inequity aversion [37–42]. For its increasingly recognized reach (e.g. [43–45]), the main focus of our piece is on *cultural* IGV, i.e. behavioural variation across groups owing to social learning within groups.

## 3. Cultural intergroup variation

Cultural IGV develops proximately as a response to ecological factors through the mechanism of social learning and can arise both between and within generations [43]. Ecological factors can be coarsely defined as all aspects of the environment that affect an organism's reproductive success. If ecological factors differ between groups of the same species, different group-specific behaviours (i.e. cultural IGV) can emerge. For instance, the absence of appropriate stone tools could prompt groups of chimpanzees to create and maintain a tradition of nut-cracking with wooden hammers, while other groups, with an abundance of stone tools in their territory, may resort to stone instead of wood technologies (cf. [16]). Conspecifics constitute a particularly influential ecological factor in social species, which comprises the complex (polyadic) interaction patterns among individuals, both within- and between groups. Owing to a constant tension between the overlapping needs of conspecifics, in conjunction with the benefits that all individuals may reap from group-living (e.g. [46,47]), behavioural phenotypes, especially in gregarious species, are opportunistically transient and as such prone to induce individual- and group-level variation. In particular, the capacity to learn socially—i.e. learning that is influenced by observation of, or interaction with, a conspecific, or its products [48]—has been identified as a source of both intraspecific variation and IGV in behavioural tendencies, not only for humans, but also for many animal species across a wide range of taxa, for instance, in birds [49], cetaceans [50], ungulates [51], insects [52] and primates [53]. Typically, when social learning is involved, IGV emerges owing to an original innovation within one particular group leading to a group-specific behavioural variant by means of within-group copying (e.g. [54]) and possibly a mechanism mitigating the eroding effects of dispersal and random drift, like conformity (e.g. [55]). Cultural IGV in behaviour can express itself both qualitatively, in terms of novel behaviours, but also quantitatively, in terms of the frequency of common behaviours. Studies of animal culture have initially focused on novel behaviours (e.g. tool-use) because the presence or absence of behaviours across groups with similar ecologies can be a salient indicator of cultural behaviour (e.g. [53]). However, groups can also differ with regard to the frequency of commonly performed behaviours (e.g. grooming, aggression) owing to culture (e.g. [12,56–58]). Observing quantitative culture requires more extensive data collection in terms of behaviour sampling within and between groups. Yet, in our view, such extended investments are worthwhile because of the potent impact quantitative culture may have on local adaptive landscapes. Culturally sustained interaction patterns can become part of individuals' selective environment [56,59], which opens up the possibility of gene–culture coevolution in animals [43,50]. For example, different killer whale ecotypes have developed distinct genetic adaptations for digesting proteins of either mammals or fish, depending on the specific cultural food preferences displayed by the respective groups over multiple generations [43,60]. This example of gene–culture coevolution in a non-human species is reminiscent of the evolution of lactase persistence in certain human populations [10] and emphasizes the importance of studying not just isolated cultural traditions in animals (e.g. nut-cracking in chimpanzees), but rather long-term patterns of social interactions *in relation to* local customs, and their potential genetic signatures [43,45,59].

## 4. Intergroup variation in chimpanzees: a synopsis

Chimpanzees show a wide variety of IGV for which several mechanisms have been identified. For instance, Western female chimpanzees (*Pan troglodytes verus*) have been reported to be more gregarious than their Eastern (*Pan troglodytes schweinfurthii*) counterparts [61], which is suggestive of the workings of genetic predispositions, although ecological factors (e.g. differing densities/probabilities of food abundances) might similarly, or even simultaneously, exert effects on local sociality [62]. Ecology has played an essential role in explaining social relationships among (especially female) primates in general [63,64]. The respective theoretical framework was coined the ‘socio-ecological model’ [63] and purported to explain social group structures by integrating ecological factors (e.g. predation risk and food abundance) with additional determinants like the risk of infanticide and habitat saturation (see e.g. [63–68]). More recently, social learning has been identified as a substantial driver of IGV in chimpanzees (i.e. cultural IGV), causing not only group-specific behavioural variants like spear hunting [69], nut-cracking [16] and handclasp-grooming [70], but possibly also substantial variation in the very fabric of within-group sociality, for instance, in terms of spatial closeness of group members (in the presence of valuable resources [71]) and grooming patterns [12] (for similar findings in other species, see: olive baboons (*Papio anubis*) [56], vervet monkeys (*Chlorocebus pygerythrus*) [14,58], sperm whales (*Physeter macrocephalus*) [72]). The perceived importance of social learning in shaping non-human primate behaviour has even increased to the extent that some scholars have proposed to integrate the capacity to learn from others into the ‘null-model’ aimed at understanding primate behaviour [73].

## 5. Reconciling scientific inconsistencies

While IGV has been acknowledged and studied by scholars working with wild chimpanzee populations (e.g. [62,74,75]), experimental studies with captive chimpanzees rarely include the possibility of IGV in their study designs and discussions. Given that experimental studies typically involve only one chimpanzee group, the tendency to avoid speculations about the influence of IGV is understandable for each single experiment. However, a systematic neglect of IGV across many studies can lead to a distorted view of what constitutes typical chimpanzee social behaviour. For instance, there is a long-standing and unresolved debate about whether chimpanzees are inequity averse or not (cf. [37–42]). Despite unavoidable differences in applied methodologies across studies (although see [38] and [42] for reporting contradictory findings with the exact same procedure), it is conceivable that chimpanzee groups may differ in their expression of inequity aversion. A hint at the possible effect of group-specific dynamics on inequity aversion was already implicit in the original study, wherein two subgroups were found to respond differently to inequitable conditions [38]. The circumstances and extent to which chimpanzees use cooperative strategies have also been debated and studies yielded mixed results [26–29]. Considering the degree of IGV with regard to social dynamics might help explain how the propensity for cooperation varies depending on certain group traits such as social tolerance [76] or steepness of hierarchies [77,78]. Similarly, the inconsistent results with respect to chimpanzees' ‘prosocial behaviour’—all acts that alleviate conspecifics' needs or improve their welfare [79]—may be an artefact of single-group studies and thus ultimately, at least partly, attributable to IGV (e.g. [30–32]). In short, the conclusions from experimental studies on chimpanzees’ prosociality range from ‘indifferent to the welfare of unrelated group members' [33] to ‘spontaneously occurring prosocial choices without solicitation’ (paraphrased from [34]). It has been shown that task-designs can influence chimpanzees' prosocial behaviour [35], but similar to the rationale of the ‘individual-differences’ approach (e.g. [36]), and in the light of the evidenced IGV in chimpanzees so far, we conjecture that chimpanzee *groups* may differ from one another in their expression of prosocial behaviour as well (cf. van Leeuwen EJC, DeTroy SE, Kaufhold SP, Dubois C, Schütte S, Call J, Haun DBM 2016, unpublished manuscript). In addition to adopting a multi-group approach, one way of testing this conjecture would be to focus on migrating individuals and assess their behavioural changes accordingly (e.g. [55,80]). Overall, we note two important considerations: (i) IGV may be more likely for expressions of *propensity* (e.g. prosociality) than for *capacity* (e.g. theory of mind)^1^ and (ii) inferences from single-group studies about species-typical behaviour need to be evaluated with caution (also see [25]). The latter consideration pertains especially to species for which substantial IGV could be envisaged. In §6, we address this issue in more detail.

## 6. How to go from here?

The perils of neglecting IGV encompass inadequate scientific scrutiny leading to premature and possibly biased ‘species-typical’ generalizations, especially in behavioural experiments that use a small sample size of subjects from the same group. In turn, such inaccurate accounts can cause artefactual inconsistencies in research findings and generate erroneous phylogenetic approximations. Beyond highlighting the need to account for cultural IGV, when a multi-group approach is not readily possible, we propose the following incremental protocol towards scientific improvement: (i) assessment of the potential for IGV in the species under study by means of literature review, (ii) interpretation of outcomes of single-group studies as representative of a specific group rather than the entire species, (iii) application of a methodologically simple assay across multiple groups within the study species, and (iv) incorporation of at least one ‘replicate’ group to validate the findings of the test-group.

With respect to suggestion (i), as a coarse heuristic, we would encourage researchers who work with species that are closely related to humans to be anticipating cultural IGV in their study species (e.g. the great apes [53,82], but also monkeys [15,83] and prosimians [84,85]). In more detail, we would recommend a literature search with the aim of finding indications for (the potential for^2^) cultural IGV in the species under study. If there are any data suggesting that groups within the study species might differ from each other in their behavioural dynamics (e.g. for birds, see [49,87]; for cetaceans, see [72,88]), the following step(s) in the protocol would be warranted, e.g. drawing inference with respect to the group instead of the entire species under study (see suggestion: (ii)). With respect to suggestion (iii), we could envisage simple measures of *social tolerance* being useful in obtaining a first indication of the possible magnitude of IGV in the species under study. Social tolerance, operationalized as the extent to which individuals within a group can be in close proximity without aggression [71], is relatively easy to assess (e.g. [71,82,89,90]) and has been reported to differ substantially across intraspecific groups [71,90]. Moreover, social tolerance forms a prerequisite for more elaborated behaviours like prosociality, cooperation and social learning (e.g. [76,82,91]), making its estimation highly relevant for obtaining a more valid ‘IGV-adjusted’ measure of species-typical behaviour. Lastly, resources permitted, the ideal scenario would be to study a substantial^3^ number of groups in a standardized fashion (see [93] for an *inter*specific approach). Lacking the means for such an encompassing project, we would encourage the assessment of at least one ‘replicate’ group (suggestion (iv)). Notwithstanding that two groups are still insufficient to reliably detect group-level effects, it may function as a proof-of-concept for the presence of IGV and provide a first estimate of its magnitude (cf. [12]).

## Endnotes

1 Although social dynamics can also mask capacities, e.g. [81].

2 Here, we refer to brain size (see [86]) and to cognitive capacities conducive to the emergence of cultural IGV, especially the social learning capacity.

3 Here, an *a priori* power analysis could be used to determine the number of groups needed to detect a group-effect with a reasonable power (e.g. [92]).

## Data accessibility

This article has no additional data.

## References

[1] Henrich J 2017 The secret of our success: how culture is driving human evolution, domesticating our species, and making us smarter. Princeton, NJ: Princeton University Press.

[2] Henrich J, Heine SJ, Norenzayan A 2010 The weirdest people in the world? Behav. Brain Sci. 33, 61–83. (10.1017/S0140525X0999152X) 20550733

[3] Nettle D 2009 Ecological influences on human behavioural diversity: a review of recent findings. Trends Ecol. Evol. 24, 618–624. (10.1016/j.tree.2009.05.013) 19683831

[4] Nielsen M, Haun D, Kärtner J, Legare CH 2017 The persistent sampling bias in developmental psychology: a call to action. J. Exp. Child Psychol. 162, 31–38. (10.1016/j.jecp.2017.04.017) 28575664 PMC10675994

[5] House BR, Silk JB, Henrich J, Barrett HC, Scelza BA, Boyette AH, Hewlett BS, McElreath R, Laurence S 2013 Ontogeny of prosocial behavior across diverse societies. Proc. Natl Acad. Sci. USA 110, 14 586–14 591. (10.1073/pnas.1221217110) PMC3767518 23959869

[6] Van Leeuwen EJC, Cohen E, Collier-Baker E, Rapold CJ, Schäfer M, Schütte S, Haun DBM 2018 The development of human social learning across seven societies. Nat. Commun. 9, 2076 (10.1038/s41467-018-04468-2) 29802252 PMC5970179

[7] Blake PR et al*.* 2015 The ontogeny of fairness in seven societies. Nature 528, 258–261. (10.1038/nature15703) 26580018

[8] Schradin C 2013 Intraspecific variation in social organization by genetic variation, developmental plasticity, social flexibility or entirely extrinsic factors. Phil. Trans. R. Soc. B 368, 20120346 (10.1098/rstb.2012.0346) 23569294 PMC3638449

[9] Kappeler PM, Barrett L, Blumstein DT, Clutton-Brock TH 2013 Constraints and flexibility in mammalian social behaviour: introduction and synthesis. Phil. Trans. R. Soc. B 368, 20120337 (10.1098/rstb.2012.0337) 23569286 PMC3638441

[10] Beja-Pereira A et al*.* 2003 Gene–culture coevolution between cattle milk protein genes and human lactase genes. Nat. Genet. 35, 311–313. (10.1038/ng1263) 14634648

[11] Hruschka DJ, Medin DL, Rogoff B, Henrich J 2018 Pressing questions in the study of psychological and behavioral diversity. Proc. Natl Acad. Sci. USA 115, 11 366–11 368. (10.1073/pnas.1814733115) PMC6233101 30397139

[12] Van Leeuwen EJC, Cronin KA, Haun DBM 2018 Population-specific social dynamics in chimpanzees. Proc. Natl Acad. Sci. USA 115, 11 393–11 400. (10.1073/pnas.1722614115) PMC6233085 30397113

[13] Cantor M, Shoemaker LG, Cabral RB, Flores CO, Varga M, Whitehead H 2015 Multilevel animal societies can emerge from cultural transmission. Nat. Commun. 6, 8091 (10.1038/ncomms9091) 26348688 PMC4569709

[14] Borgeaud C, Sosa S, Bshary R, Sueur C, van de Waal E 2016 Intergroup variation of social relationships in wild vervet monkeys: a dynamic network approach. Front. Psychol. 7, 915 (10.3389/fpsyg.2016.00915) 27445890 PMC4914564

[15] Santorelli CJ, Schaffner CM, Campbell CJ, Notman H, Pavelka MS, Weghorst JA, Aureli F 2011 Traditions in spider monkeys are biased towards the social domain. PLoS ONE 6, e16863 (10.1371/journal.pone.0016863) 21373196 PMC3044143

[16] Luncz LV, Mundry R, Boesch C 2012 Evidence for cultural differences between neighboring chimpanzee communities. Curr. Biol. 22, 922–926. (10.1016/j.cub.2012.03.031) 22578420

[17] Bolnick DI et al*.* 2011 Why intraspecific trait variation matters in community ecology. Trends Ecol. Evol. 26, 183–192. (10.1016/j.tree.2011.01.009) 21367482 PMC3088364

[18] Lott DF 1984 Intraspecific variation in the social systems of wild vertebrates. Behaviour 88, 266–325. (10.1163/156853984X00353)

[19] Sapolsky RM 2017 Behave: the biology of humans at our best and worst. New York, NY: Penguin.

[20] Fashing PJ 2001 Activity and ranging patterns of guerezas in the Kakamega Forest: intergroup variation and implications for intragroup feeding competition. Int. J. Primatol. 22, 549–577. (10.1023/A:1010785517852)

[21] Brotcorne F, Giraud G, Gunst N, Fuentes A, Wandia IN, Beudels-Jamar RC, Poncin P, Huynen MC, Leca JB 2017 Intergroup variation in robbing and bartering by long-tailed macaques at Uluwatu Temple (Bali, Indonesia). Primates 58, 505–516. (10.1007/s10329-017-0611-1) 28516338

[22] Hopper LM, Schapiro SJ, Lambeth SP, Brosnan SF 2011 Chimpanzees' socially maintained food preferences indicate both conservatism and conformity. Anim. Behav. 81, 1195–1202. (10.1016/j.anbehav.2011.03.002) 27011390 PMC4801479

[23] Koops K, Schöning C, Isaji M, Hashimoto C 2015 Cultural differences in ant-dipping tool length between neighbouring chimpanzee communities at Kalinzu, Uganda. Sci. Rep. 5, e12456 (10.1038/srep12456) PMC4510480 26198006

[24] Luncz LV, Boesch C 2014 Tradition over trend: neighboring chimpanzee communities maintain differences in cultural behavior despite frequent immigration of adult females. Am. J. Primatol. 76, 649–657. (10.1002/ajp.22259) 24482055

[25] Strier KB 2009 Seeing the forest through the seeds. Curr. Anthropol. 50, 213–228. (10.1086/592026) 19817224

[26] Bullinger AF, Melis AP, Tomasello M 2011 Chimpanzees, *Pan troglodytes*, prefer individual over collaborative strategies towards goals. Anim. Behav. 82, 1135–1141. (10.1016/j.anbehav.2011.08.008)

[27] Suchak M, Eppleya TM, Campbell MW, Feldmana RA, Quarlesc LF, De Waal FBM 2016 How chimpanzees cooperate in a competitive world. Proc. Natl Acad. Sci. USA 113, 10 215–10 220. (10.1073/pnas.1611826113) 27551075 PMC5018789

[28] Schmidt MFH, Tomasello M 2016 How chimpanzees cooperate: if dominance is artificially constrained. Proc. Natl Acad. Sci. USA 113, E6728–E6729. (10.1073/pnas.1614378113) 27791161 PMC5098606

[29] Suchak M, De Waal FBM 2016 Reply to Schmidt and Tomasello: Chimpanzees as natural team-players. Proc. Natl Acad. Sci. USA 113, E6730 (10.1073/pnas.1614598113) 27791163 PMC5098646

[30] Jensen K, Tennie C, Call J 2018 Correspondence: Reply to ‘Chimpanzee helping is real, not a byproduct’. Nat. Commun. 9, 616 (10.1038/s41467-017-02328-z) 29434268 PMC5809583

[31] Tennie C, Jensen K, Call J 2016 The nature of prosociality in chimpanzees. Nat. Commun. 7, 13915 (10.1038/ncomms13915) 27996969 PMC5187495

[32] Melis AP, Engelmann JM, Warneken F 2018 Correspondence: Chimpanzee helping is real, not a byproduct. Nat. Commun. 9, 616 (10.1038/s41467-017-02321-6) 29434291 PMC5809601

[33] Silk JB, Brosnan SF, Vonk J, Henrich J, Povinelli DJ, Richardson AS, Lambeth SP, Mascaro J, Schapiro SJ 2005 Chimpanzees are indifferent to the welfare of unrelated group members. Nature 437, 1357–1359. (10.1038/nature04243) 16251965

[34] Horner V, Carter JD, Suchak M, De Waal FBM 2011 Spontaneous prosocial choice by chimpanzees. Proc. Natl Acad. Sci. USA 108, 13 847–13 851. (10.1073/pnas.1111088108) PMC3158226 21825175

[35] House BR, Silk JB, Lambeth SP, Schapiro SJ 2014 Task design influences prosociality in captive chimpanzees (*Pan troglodytes*). PLoS ONE 9, e0103422 (10.1371/journal.pone.0103422) PMC4156467 25191860

[36] Rosati AG, DiNicola LM, Buckholtz JW 2018 Chimpanzee cooperation is fast and independent from self-control. Psychol. Sci. 29, 1832–1845. (10.1177/0956797618800042) 30295562

[37] Brosnan SF, Talbot C, Ahlgren M, Lambeth SP, Schapiro SJ 2010 Mechanisms underlying responses to inequitable outcomes in chimpanzees, *Pan troglodytes*. Anim. Behav. 79, 1229–1237. (10.1016/j.anbehav.2010.02.019) 27011389 PMC4801319

[38] Brosnan SF, Schiff HC, De Waal FBM 2005 Tolerance for inequity may increase with social closeness in chimpanzees. Proc. R. Soc. B 272, 253–258. (10.1098/rspb.2004.2947) PMC1634968 15705549

[39] Kim Y, Choe JC, Jeong G, Kim D, Tomonaga M 2018 Chimpanzees but not orangutans display aversive reactions toward their partner receiving a superior reward. *bioRxiv* 274803 (10.1101/274803)

[40] Ulber J, Hamann K, Tomasello M 2017 Young children, but not chimpanzees, are averse to disadvantageous and advantageous inequities. J. Exp. Child Psychol. 155, 48–66. (10.1016/j.jecp.2016.10.013) 27918977

[41] Bräuer J, Call J, Tomasello M 2006 Are apes really inequity averse? Proc. R. Soc. B 273, 3123–3128. (10.1098/rspb.2006.3693) PMC1679898 17015338

[42] Bräuer J, Call J, Tomasello M 2009 Are apes inequity averse? New data on the token-exchange paradigm. Am. J. Primatol. 71, 175–181. (10.1002/ajp.20639) 19021260

[43] Whitehead H, Laland KN, Rendell L, Thorogood R, Whiten A 2019 The reach of gene–culture coevolution in animals. Nat. Commun. 10, 2405 (10.1038/s41467-019-10293-y) 31160560 PMC6546714

[44] Whiten A, Ayala FJ, Feldman MW, Laland KN 2017 The extension of biology through culture. Proc. Natl Acad. Sci. USA 114, 7775–7781. (10.1073/pnas.1707630114) 28739924 PMC5544333

[45] Whiten A 2019 Cultural evolution in animals. Annu. Rev. Ecol. Evol. Syst. 50 (10.1146/annurev-ecolsys-110218-025040)

[46] Van Schaik CP 1983 Why are diurnal primates living in groups? Behaviour 87, 120–144. (10.1163/156853983X00147)

[47] Ward A, Webster M 2016 Sociality: the behaviour of group-living animals. Berlin, Germany: Springer.

[48] Heyes CM 1994 Social learning in animals: categories and mechanisms. Biol. Rev. Camb. Philos. Soc. 69, 207–231. (10.1111/j.1469-185X.1994.tb01506.x) 8054445

[49] Aplin LM, Farine DR, Morand-Ferron J, Cockburn A, Thornton A, Sheldon BC 2015 Experimentally induced innovations lead to persistent culture via conformity in wild birds. Nature 518, 538–541. (10.1038/nature13998) 25470065 PMC4344839

[50] Whitehead H 2017 Gene–culture coevolution in whales and dolphins. Proc. Natl Acad. Sci. USA 114, 7814–7821. (10.1073/pnas.1620736114) 28739936 PMC5544266

[51] Jesmer BR et al*.* 2018 Is ungulate migration culturally transmitted? Evidence of social learning from translocated animals. Science 361, 1023–1025. (10.1126/science.aat0985) 30190405

[52] Danchin E et al*.* 2018 Cultural flies: conformist social learning in fruitflies predicts long-lasting mate-choice traditions. Science 362, 1025–1030. (10.1126/science.aat1590) 30498121

[53] Whiten A, Goodall J, McGrew WC, Nishida T, Reynolds V, Sugiyama Y, Tutin CEG, Wrangham RW, Boesch C 1999 Cultures in chimpanzees. Nature 399, 682–685. (10.1038/21415) 10385119

[54] van Leeuwen EJC, Cronin KA, Haun DBM 2014 A group-specific arbitrary tradition in chimpanzees (*Pan troglodytes*). Anim. Cogn. 17, 1421–1425. (10.1007/s10071-014-0766-8) 24916739

[55] Van De Waal E, Borgeaud C, Whiten A 2013 Potent social learning and conformity shape a wild primate's foraging decisions. Science 340, 483–485. (10.1126/science.1232769) 23620053

[56] Sapolsky RM, Share LJ 2004 A pacific culture among wild baboons: its emergence and transmission. PLoS Biol. 2, e106 (10.1371/journal.pbio.0020106) 15094808 PMC387274

[57] Sapolsky RM 2006 Social cultures among nonhuman primates. Curr. Anthropol. 47, 641–656. (10.1086/504162)

[58] van de Waal E 2018 On the neglected behavioural variation among neighbouring primate groups. Ethology 124, 845–854. (10.1111/eth.12815)

[59] Cantor M, Whitehead H 2013 The interplay between social networks and culture: theoretically and among whales and dolphins. Phil. Trans. R. Soc. B 368, 20120340 (10.1098/rstb.2012.0340) 23569288 PMC3638443

[60] Foote AD et al*.* 2016 Genome-culture coevolution promotes rapid divergence of killer whale ecotypes. Nat. Commun. 7, 11693 (10.1038/ncomms11693) 27243207 PMC4895049

[61] Lehmann J, Boesch C 2008 Sexual differences in chimpanzee sociality. Int. J. Primatol. 29, 65–81. (10.1007/s10764-007-9230-9) 19816541 PMC2758377

[62] Wrangham RW, de Waal FBM, McGrew WC 1994 The challenge of behavioral diversity. In Chimpanzee cultures (eds Wrangham RW, McGrew WC, de Waal FBM, Heltne PG), pp. 1–19. Cambridge, MA: Harvard University Press.

[63] Sterck EHM, Watts DP, Van Schaik CP 1997 The evolution of female social relationships in nonhuman primates. Behav. Ecol. Sociobiol. 41, 291–309. (10.1007/s002650050390)

[64] Wrangham RW 1980 An ecological model of female-bonded primate groups. Behaviour 75, 262–300. (10.1163/156853980X00447)

[65] Van Schaik CP 1989 The ecology of social relationships amongst female primates. In Comparative socioecology: the behavioural ecology of humans and other mammals (eds Standen V, Foley RA), pp. 195–218. Oxford, UK: Blackwell Scientific.

[66] Schulke O, Ostner J 2012 Ecological and social influences on sociality. In The evolution of primate societies (eds Mitani JC, Call J, Kappeler PM, Palombit RA, Silk JB), pp. 195–219. Chicago, IL: University of Chicago Press.

[67] Thierry B 2008 Primate socioecology, the lost dream of ecological determinism. Evol. Anthropol. 17, 93–96. (10.1002/evan.20168)

[68] Clutton-Brock T, Janson C 2012 Primate socioecology at the crossroads: past, present, and future. Evol. Anthropol. 21, 136–150. (10.1002/evan.21316) 22907867

[69] Pruetz JD, Bertolani P 2007 Savanna chimpanzees, *Pan troglodytes verus*, hunt with tools. Curr. Biol. 17, 412–417. (10.1016/j.cub.2006.12.042) 17320393

[70] van Leeuwen EJC, Cronin KA, Haun DBM, Mundry R, Bodamer MD 2012 Neighbouring chimpanzee communities show different preferences in social grooming behaviour. Proc. R. Soc. B 279, 4362–4367. (10.1098/rspb.2012.1543) PMC3479803 22933372

[71] Cronin KA, van Leeuwen EJC, Vreeman V, Haun DBM 2014 Population-level variability in the social climates of four chimpanzee societies. Evol. Hum. Behav. 35, 389–396. (10.1016/j.evolhumbehav.2014.05.004)

[72] Cantor M, Whitehead H 2015 How does social behavior differ among sperm whale clans? Mar. Mammal Sci. 31, 1275–1290. (10.1111/mms.12218)

[73] Van Schaik C, Graber S, Schuppli C, Burkart J 2017 The ecology of social learning in animals and its link with intelligence. Span. J. Psychol. 19, E99 (10.1017/sjp.2016.100) 28065213

[74] Whiten A, Goodall J, McGrew WC, Nishida T, Reynolds V, Sugiyama Y, Tutin CEG, Wrangham RW, Boesch C 2001 Charting cultural variation in chimpanzees. Behaviour 138, 1481–1516. (10.1163/156853901317367717)

[75] Boesch C 2012 Wild cultures: a comparison between chimpanzee and human cultures. Cambridge, UK: Cambridge University Press.

[76] Melis AP, Hare B, Tomasello M 2006 Engineering cooperation in chimpanzees: tolerance constraints on cooperation. Anim. Behav. 72, 275–286. (10.1016/j.anbehav.2005.09.018)

[77] Kaburu SSK, Newton-Fisher NE 2015 Egalitarian despots: hierarchy steepness, reciprocity and the grooming-trade model in wild chimpanzees, *Pan troglodytes*. Anim. Behav. 99, 61–71. (10.1016/j.anbehav.2014.10.018) PMC4287234 25580017

[78] Jaeggi AV, Stevens JMG, Van Schaik CP 2010 Tolerant food sharing and reciprocity is precluded by despotism among bonobos but not chimpanzees. Am. J. Phys. Anthropol. 143, 41–51. (10.1002/ajpa.21288) 20310060

[79] Cronin KA 2012 Prosocial behaviour in animals: the influence of social relationships, communication and rewards. Anim. Behav. 84, 1085–1093. (10.1016/j.anbehav.2012.08.009)

[80] Vale GL, Davis SJ, van de Waal E, Schapiro SJ, Lambeth SP, Whiten A 2017 Lack of conformity to new local dietary preferences in migrating captive chimpanzees. Anim. Behav. 124, 135–144. (10.1016/j.anbehav.2016.12.007) 29200465 PMC5705092

[81] Drea CM, Wallen K 1999 Low-status monkeys ‘play dumb’ when learning in mixed social groups. Proc. Natl Acad. Sci. USA 96, 12 965–12 969. (10.1073/pnas.96.22.12965) PMC23184 10536031

[82] Van Schaik CP, Ancrenaz M, Borgen G, Galdikas B, Knott CD, Singleton I, Suzuki A, Suci Utami S, Merrill M 2003 Orangutan cultures and the evolution of material culture. Science 299, 102–105. (10.1126/science.1078004) 12511649

[83] Perry S et al*.* 2003 Social conventions in wild white-faced capuchin monkeys: evidence for traditions in a neotropical primate. Curr. Anthropol. 44, 241–268. (10.1086/345825)

[84] Kendal RL, Custance DM, Kendal JR, Vale G, Stoinski TS, Rakotomalala NL, Rasamimanana H 2010 Evidence for social learning in wild lemurs (*Lemur catta*). Learn. Behav. 38, 220–234. (10.3758/LB.38.3.220) 20628161

[85] Stoinski TS, Drayton LA, Price EE 2011 Evidence of social learning in black-and-white ruffed lemurs (*Varecia variegata*). Biol. Lett. 7, 376–379. (10.1098/rsbl.2010.1070) 21227976 PMC3097868

[86] van Schaik CP 2013 The costs and benefits of flexibility as an expression of behavioural plasticity: a primate perspective. Phil. Trans. R. Soc. B 368, 20120339 (10.1098/rstb.2012.0339) 23569287 PMC3638442

[87] Mundinger PC 1982 Microgeographic and macrogeographic variation in birds. In Acoustic communication in birds (eds Kroodsma DE, Miller EH, Oullet H), pp. 147–208. New York, NY: Academic Press.

[88] Krützen M, Mann J, Heithaus MR, Connor RC, Bejder L, Sherwin WB 2005 Cultural transmission of tool use in bottlenose dolphins. Proc. Natl Acad. Sci. USA 102, 8939–8943. (10.1073/pnas.0500232102) 15947077 PMC1157020

[89] Horn L, Scheer C, Bugnyar T, Massen JJM 2016 Proactive prosociality in a cooperatively breeding corvid, the azure-winged magpie (*Cyanopica cyana*). Biol. Lett. 12, 20160649 (10.1098/rsbl.2016.0649) 28120800 PMC5095199

[90] Fichtel C, Schnoell AV, Kappeler PM 2018 Measuring social tolerance: an experimental approach in two lemurid primates. Ethology 124, 65–73. (10.1111/eth.12706)

[91] Hare B, Melis AP, Woods V, Hastings S, Wrangham R 2007 Tolerance allows bonobos to outperform chimpanzees on a cooperative task. Curr. Biol. 17, 619–623. (10.1016/j.cub.2007.02.040) 17346970

[92] Cohen J 2013 Statistical power analysis for the behavioral sciences. Abingdon-on-Thames, UK: Routledge.

[93] Burkart JM et al*.* 2014 The evolutionary origin of human hyper-cooperation. Nat. Commun. 5, 4747 (10.1038/ncomms5747) 25158760

